# Supplementary figures and images for: Selenite Downregulates STAT3 Expression and Provokes Lymphocytosis in the Liver of Chronically Exposed Syrian Golden Hamsters
Source: Molecules. 2021 Sep 16;26(18):5614. doi: 10.3390/molecules26185614 (PMC8465886; doi:10.3390/molecules26185614)

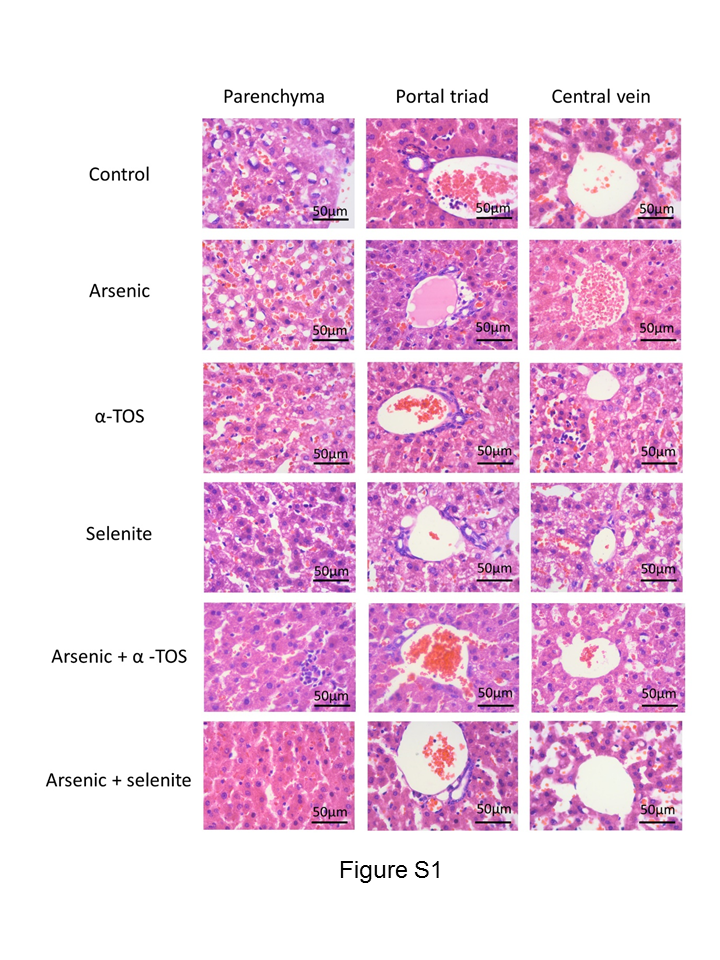

Supplement: Supplementary file 1 [file molecules-26-05614-s001.zip › molecules-1311476-supplementary.tif]
